# Characterization of Internet Gaming Addiction among School Children: A Cross-Sectional Study

**DOI:** 10.2174/0117450179451710260206215203

**Published:** 2026-02-18

**Authors:** Suzie Rababa’h, Karem H. Alzoubi, Rania Mahafdeh, Iman Basheti, Zahra A. Sayed, Ahmed Alhusban

**Affiliations:** 1 Department of Pharmacy, Faculty of Pharmacy, Jadara University, Irbid, Jordan; 2 Department of Pharmaceutical Sciences, College of Pharmacy, QU Health, Qatar University, Doha, Qatar; 3 Department of Respiratory Therapy, Faculty of Allied Medical Sciences, Jadara University, Irbid, Jordan; 4 Department of Pharmacy Practice and Pharmacotherapeutics, College of Pharmacy, University of Sharjah, Sharjah, UAE; 5 Department of Clinical Pharmacy, Faculty of Pharmacy, Jordan University of Science and Technology, Irbid, Jordan

**Keywords:** Children, Video games, Teenagers, Preteens, Academic achievement, Developing nation

## Abstract

**Introduction:**

Targeting the younger generation and growing appeal among older users, video games have become an important and timely source of entertainment for teenagers and preteens. The study aims to assess the relationship between Jordanian schoolchildren's academic success and online gaming addiction.

**Methods:**

A sample of 458 children, whose ages were 9 to 17, were randomly chosen from five private schools to participate in this cross-sectional survey, which was carried out in Jordan between May and July 2024. Pre-teens (9–12 years old) and adolescents (13–17 years old) were the two groups of participants. The Internet Gaming Disorder Scale–Short-Form (IGDS9-SF) in Arabic was used to measure the severity of gaming. Self-reported academic achievement, gaming habits, and sociodemographic information were all covered *via* a standardized questionnaire. The t-test and chi-square test were used to analyse group differences; *p* < 0.05 was deemed statistically significant.

**Results:**

The findings indicated that adolescents' excessive gaming was more severe than that of preteens. Teens reported a poor correlation with their academic performance, were more likely to use gaming apps during class, and had trouble focusing. Teens, who made up 63.5% of the participants, reported a higher perceived negative impact of gaming applications on their overall GPA that did not reach statistical significance. The overall effect of gaming on meeting deadlines and exam preparation did not differ significantly between age groups. Internet gaming scores were high among teenagers (*p* < 0.05). Furthermore, compared to pre-teens, teenagers reported using the internet for gaming more frequently in class (*p*= 0.049). Teens also claimed that using gaming applications negatively affected their ability to focus in class (*p*<0.05).

**Discussion:**

The findings showed that excessive usage of gaming apps in class has a more detrimental effect on students' ability to concentrate, but it has no significant effect on missing deadlines or preparing for exams. These findings highlight the significance of keeping an eye on gaming behaviour in order to reduce the negative effects of IGD on concentration in the classroom.

**Conclusion:**

Video game disorder is quite prevalent among Jordanian private school students.These results underscore the need for more long-term studies using objective academic indicators and larger samples to elucidate the educational implications of internet gaming in this population. They also show that attention during class and difficulty focusing during lessons are regarded as potential areas of concern in the context of intensive gaming.

## 1 INTRODUCTION

A big part of childhood these days is playing video games. Children's main motivations for playing video games are enjoyment, competitiveness, and social interaction [1]. The computer video game industry brought in around 450 billion US dollars worldwide in 2024 [2]. Most children and adolescents between the ages of 8 and 17 spend on average two hours a day gaming and are actively integrating gaming into their lives, according to a recent study [3, 4]. Video games may improve children's lives and lessen stress, according to some studies [5]. However, adolescents and teens who are particularly sensitive may experience serious negative consequences from excessive computer gaming [2-4].

These studies support the growing global prevalence of video game usage among kids and teenagers, with data showing that many kids engage in gaming daily [2-4]. In addition to the significant growth in the global video game market, it confirms the considerable impact of games on modern society. In addition, studies have highlighted the potentially harmful outcomes among young people, often associated with gaming addiction and tolerance, such as aggressive cognition and behavior, emotional problems such as depression, poor academic achievement, and inattention [1, 2].

World Health Organization (WHO), in the International Classification of Diseases, eleventh revision (ICD-11), defined problematic video gaming disorder as an uncontrollable and persistent gaming behavior leading to a significant impairment in personal, social, occupational, or other important areas of functioning [6]. The American Psychiatric Association, in the Diagnostic and Statistical Manual of Mental Disorders, 5th edition (DSM-5), considered problematic internet gaming as a potential disorder of non-substance addictive behaviors that needs further clinical research before inclusion as a formal disorder [7]. In recent years, IGD (whether online or offline) has become a worldwide issue, and the prevalence of teenage video game addiction is rising [2]. Young people addicted to video games feel compelled to keep playing [8, 9]. Teens and children may be especially vulnerable to gaming's negative impacts if they struggle with social skills, low self-esteem, boredom, loneliness, learning difficulties, or mental health issues. The issue arises when video games excel at something; they fulfill psychological requirements like the drive to feel autonomous, forget about issues in the real world, and escape [4]. While some researchers suggest that exposure to video games may negatively impact kids, the overall effects on their psychosocial development remain highly debated and need more research [10].

IGD prevalence varies widely between nations, with Eastern Asia thought to have the highest frequency. Adolescent boys between the ages of 12 and 20 had the highest rates worldwide [2]. There are not many studies on children's video game addiction in Jordan. Jordanian parents indicated that their children between the ages of 4 and 10 spend an average of 7 h during the weekdays playing video games in a single recent research [11]. However, no research has looked at the relationship between academic success and video game addiction in Jordan. Research on IGD's mental comorbidities and how they are directly linked to negative outcomes, including poor sleep, a higher likelihood of depression symptoms, anxiety, and poor academic performance, is expanding quickly [12-14].

However, a large portion of this study has mostly concentrated on adult viewpoints, which raises questions regarding the reliability and generalizability of findings to younger populations. The study investigated the prevalence and characteristics of video game use among schoolchildren, who are between the ages of 9 and 17, in Jordan. This is due to the rising prevalence of video game use among adolescents and growing concerns about its potential negative effects, including the recognition of IGD as a formal disorder. The study advances knowledge of the type and scope of video game use and its possible effects on adolescents' well-being. This knowledge is crucial for creating successful interventions to address potential risks related to excessive gaming among Jordanian adolescents.

## 2 METHODS

### 2.1 Design

The data was collected in 2024 (between May and July). The students' parents received the questionnaire, which included a brief explanation of the study, guarantees about data security, and consent. The study was authorized by Jadara University's Institutional Review Board (approval number: PHARM-JA-5/2024). From all parents and legal guardians, verbal assent was obtained from participating children in age-appropriate language, in accordance with the Jadara University IRB requirements, the Declaration of Helsinki, and international guidelines (WHO/APA) for research involving vulnerable participants. The study was conducted in accordance with the Strengthening the Reporting of Observational Studies in Epidemiology (STROBE) reporting guideline for cross-sectional studies.

### 2.2 Participants

Students were selected at random from the list provided by the school administration. Participation was open to all children aged 9 to 17. The pupils' parents responded to the survey. Parents were informed about the study's goals and procedures before participating and assured that their involvement would remain anonymous. Parents were not compensated financially for participating in the study and were free to accept or reject it.

**Fig. (1):**
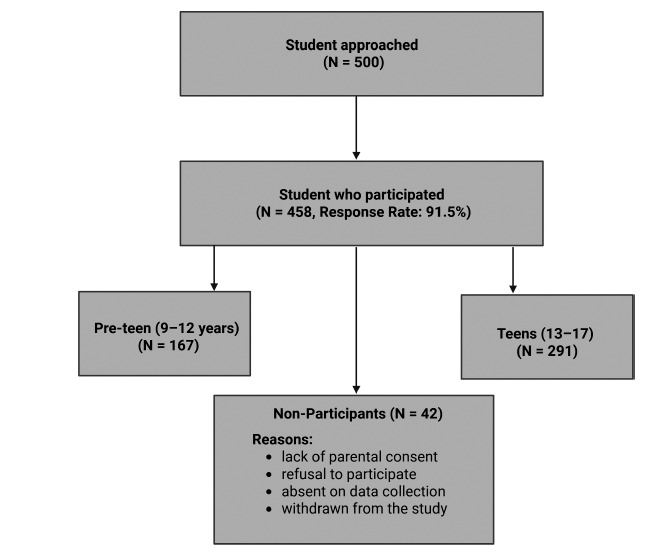
The flow chart of the recruitment process of the participants.

There were two participant groups: Group 1 (Pre-teen): 9–12-year-old students; Group 2 (Teen): 13–17-year-old students. Figure **1** shows the participant recruitment process, response rate, and reasons for non-participation.

### 2.3 Inclusion and Exclusion Criteria

All students aged 9–17 years who were enrolled in the selected private schools in Jordan during the study period (May–July 2024) were eligible for inclusion. Students were included if they provided complete responses to the study questionnaire and their parents or guardians provided written informed consent, with verbal assent from the students themselves. Students were excluded if they: Did not obtain parental consent or personal assent; submitted incomplete or invalid questionnaires (*e.g*., missing more than 10% of items); or reported a history of diagnosed psychiatric or neurological disorders that could confound Internet Gaming Disorder (IGD) assessment.

### 2.4 The Sample Size Calculation

Cochran's Formula for cross-sectional research was used to get the sample size: n = Z^2^ P (1-P)/d^2^. P is the estimated prevalence of Internet Gaming Disorder (IGD) with a 5% margin of error and a 95% confidence level. We used previous regional research to estimate P, including a recent study that found that 15.2% of Jordanian university students had IGD [15]. However, a frequency of 20.45% was discovered in a sample of Saudi teenagers [16]. We cautiously assumed an estimated prevalence of 20% due to the variation in prevalence rates among age groups. The equation suggested a minimum sample size of 246 people.

We ultimately recruited 458 students from five randomly chosen private schools to ensure sufficient representation across various age groups (pre-teens and teens) and minimize selection bias in order to increase statistical power and account for potential missing or incomplete responses. A total of 458 kids were chosen at random from five different schools; 167 of them were preteens, with 64 (33.7%) being female and 103 (38.4%) being male. Of the 291 teenagers that took part, 126 (66.3%) were female, and 165 (61.6%) were male.

### 2.5 Instruments and Variables

The study included the following variables and instruments: sociodemographic information and video gaming behaviours. Questions about the features of gaming app usage and their overall effect on academic achievement were included in the survey.

The Arabic version of the Internet Gaming Disorder Scale–Short-Form (IGDS9-SF) was used to assess the degree of use of online gaming applications. Based on the nine-item criteria recommended by the Diagnostic and Statistical Manual of Mental Disorders, Fifth Edition (DSM-5), the IGDS-9SF is a validated instrument. This scale has a maximum value of 45 points and a range of 9.

### 2.6 Analysis of Statistics

Means and standarddeviations (SDs) were used to represent continuous variables, such as age and Internet Gaming Disorder Scale–Short-Form (IGDS9-SF) scores. Frequencies and percentages were used to display categorical characteristics like gender and gaming habits. Student's t-test for continuous variables and chi-square tests for categorical variables were used to compare pre-teens (9–12 years) and adolescents (13–17 years). Based on recognised developmental phases and taking into account the variations in cognitive and behavioural development between pre-teens and adolescents, the participants were divided into these age groups. The impact magnitude of the difference between the scores of adolescents and preteens was evaluated using Cohen's d test. The pooled SD calculation was used since the group sizes were not equal. Before analysis, missing data were evaluated.

A participant's responses were eliminated from the research if they were absent for important factors (such as academic achievement indicators or IGDS9-SF scores). The SPSS version 24 (IBM SPSS Statistics for Windows, Version 24.0), Armonk, NY: IBM Corp., was used to analyse data. A *p*-value of less than 0.05 was considered statistically significant.

## 3 RESULTS

### 3.1 Demographic Distribution by Age Category

Participants were categorized into 167 pre-teens (9–12 years) and 291 teens (13–17 years). Table **1** presents the demographic distribution by age category. The total number of pre-teens is 167, with 103 males (61.6%) and 64 females (38.4%). Among the 291 teens participating in this research, 165 (56.7%) were male, and 126 (43.3%) were female. In terms of accommodation, most of both pre-teens and teens resided with their families, accounting for 165 (98.8%) and 289 (99.3%), respectively. Only two individuals from each age category were accommodated in dorms, and none of the participants resided in privately owned housing. The extent of internet gaming use was significantly greater among teens as compared to preteens (28.2 *vs*. 32; *p*=0.001) with a Cohen’s d score of 0.42, which shows a moderate-sized influence.

### 3.2 Characteristics of Gaming Applications Used according to Age Categories

An examination of the features of gaming application usage by age group is shown in Table **2**, which yields a number of important conclusions. Pre-teens and teenagers did not substantially differ in how many gaming apps they utilized daily (*p* = 0.257). Specifically, 35.2% of pre-teens and 64.8% of teens reported not using any gaming applications daily. Meanwhile, 37.8% of pre-teens and 62.2% of teens reported using more than five gaming applications daily. However, a significant difference was observed in gaming application usage during class (*p* = 0.049). Among pre-teens, 35.5% reported never using gaming applications during class, compared to 64.5% of teens. The usage patterns for rarely, sometimes, often, and always using gaming applications during class were 45.3%, 37.8%, 53.3%, and 6.7% for pre-teens, respectively, compared to 54.7%, 62.2%, 46.7%, and 93.3% for teens.

There was no difference between the age groups in the effect of using gaming applications on meeting deadlines (*p* = 0.719). Among pre-teens, 34.5% reported no impact, 34.3% reported rarely being impacted, 42.1% sometimes, 38.2% often, and 33.3% always, compared to 65.5%, 65.7%, 57.9%, 61.8%, and 66.7% of teens, respectively. Similarly, the impact on exam preparation did not differ significantly (*p* = 0.179). Among pre-teens, 35.0% reported no effect, compared to 65.0% of teens. Rare impacts were reported by 44.8% of pre-teens and 55.2% of teens. Sometimes, impacts were noted by 37.6% of pre-teens and 62.4% of teens. Often, impacts were reported by 25.0% of pre-teens and 75.0% of teens. Impacts were reported by 25.0% of pre-teens and 75.0% of teens.

The ability to attend the first lesson the next day after waking up reached significance (*p* = 0.070). Among pre-teens, 32.8% reported no impact, 44.3% reported rarely being impacted, 45.6% sometimes, 31.4% often, and 24.1% always, compared to 67.2%, 55.7%, 54.4%, 68.6%, and 75.9% of teens, respectively. A significant difference was found in the impact on students' ability to focus during class (*p* = 0.003). For pre-teens, 34.6% reported no effect, 32.0% reported rarely being impacted, 55.6% sometimes, 33.3% often, and 15.8% always, compared to 65.4%, 68.0%, 44.4%, 66.7%, and 84.2% of teens, respectively.

The frequency of teachers asking students to stop using electronic games during classes or practical sessions showed a significant difference (*p* = 0.00). Among pre-teens, 31.8% reported never being asked to stop, 42.9% rarely, 51.5% sometimes, 90.0% often, and 87.5% always, compared to 68.2%, 57.1%, 48.5%, 10.0%, and 12.5% of teens, respectively.

Perceptions of the negative impact of gaming applications did not differ significantly between the age groups (*p* = 0.364). Among pre-teens, 39.4% reported no perceived negative impacts, 38.1% reported rarely, 38.0% sometimes, 25.0% often, and 30.8% always, compared to 60.6%, 61.9%, 62.0%, 75.0%, and 69.2% of teens, respectively.

## 4 DISCUSSION

To our knowledge, this study is the first to examine self-reported internet gaming behavior among school-children in Jordan using the IGD-related scale. The findings indicate that teens reported higher IGDS0-SF scores and more frequent in-class gaming than pre-teens. Additionally, there was no statistically significant correlation found between gaming behaviour and meeting deadlines, test preparation, or GPA, although it was linked to problems concentrating in class.

Self-reported measures measuring students' ability to fulfil deadlines, get ready for tests, focus in class, wake up, and attend the first class were used to investigate academic-related outcomes in our study. Instead of being utilized as a diagnostic instrument to identify IGD cases or gauge severity, the Arabic version of the IGDS9-SF was employed as a continuous measure of gaming-related issue severity. The findings of this study have a number of significant implications for comprehending and resolving classroom engagement and school function.

The findings showed that there was no meaningful correlation between overall academic achievement and the intensity of gaming. Nonetheless, strong correlations between students' gaming habits and their inability to concentrate in class have been demonstrated, highlighting classroom attentiveness as an area that is susceptible to the gaming pattern. Another significant finding was the relationship between age and the characteristics of gaming application usage. The frequency of daily gaming application use did not change significantly between pre-teens and adolescents; there were evident variations in specific behaviours. We noticed no obvious difference in male and female teens' daily gaming application usage. However, when it came to playing gaming applications in class, there was a significant difference (*p* = 0.049).

Adolescents were more likely than preteens to use gaming apps on a regular or consistent basis. This suggests that teens may be more readily distracted when doing academic activities, affecting their capacity to concentrate and learn. Compared to 64.5% of teenagers, 35.5% of preteens stated that they had never utilised gaming applications in class. With almost significant results (*p* = 0.070), this study provides persuasive evidence that video game addiction is related to students' ability to wake up the next day and attend their first class. Teens were more likely to report negative effects in both areas, indicating that excessive use of gaming applications might interfere with attention and sleep habits. Preteens were far more likely than other students to be asked to stop using gaming apps during lectures or practical sessions.

This implies that although both age groups may play video games in class, teens are more likely to be detected or seen as interfering with the educational process. Furthermore, a significant difference in the effect of gaming on students' capacity to concentrate in class was found in the current data (*p* = 0.003). Several international studies are consistent with our results. Ward (2018) reported that as video game use increases, some students who spend more time playing, skip school, or are less diligent with their homework [17]. Other research from Turkey showed that self-regulation abilities and the negative automatic thoughts scale had a mediating role between addiction to video games and the reasoning skills of middle school students [18]. A cross-sectional study from Pakistan demonstrated that IGD was associated with poorer academic performance, specifically among adolescent males [19]. A large cross-sectional and meta-analysis study demonstrated that overuse of video gaming may impair academic performance among school students and is associated with psychological difficulties such as stress, anxiety, and depression [20-22]. A recent systematic review by Alzahrani and Griffiths (2024) demonstrated a negative association between problematic gaming and academic performance, but they also demonstrated that most available studies are cross-sectional, which cannot establish the direction of the effect [23]. Even though these findings are well known worldwide, further debate is needed to clarify their implications within local and regional settings, particularly in the Middle East [24].

In our region, Farchakh *et al*. (2020) showed a strong negative correlation between higher levels of video game addiction and attention among Lebanese schoolchildren. Higher attention score and worse memory, cognitive, and academic abilities are closely associated with a higher risk of video game addiction [25]. According to a cross-sectional study conducted in Qatar [1] IGD is more common among schoolchildren, with a prevalence of around 8.6%, especially in male students compared to female students. In Jordan, Al-Ali *et al* (2018) assessed parents' awareness of their children's exposure to media and video games between the ages of 6 and 11. 63.7% of the parents reported that their children spent around three hours a day playing video games. The findings revealed that children who spend more time playing video games are more likely to show aggressive behavior [26].

However, there is no data about the relationship between IGD and academic achievement, and studies that have examined children's self-reported video gaming behaviours in Jordan are rare.

Our results highlight how crucial it is for parents and educators to understand the possible drawbacks of excessive gaming application use. Even though gaming may provide social engagement, it is crucial to make sure it does not conflict with scholastic obligations and well-being. Potential negative impacts can be lessened by implementing techniques, including setting time limits for gaming and encouraging sound sleeping practices.

To fully comprehend the connection between the use of gaming apps and academic achievement, more study is needed. Future research could examine the kinds of gaming applications that have the biggest effects on learning outcomes, the influence of parental supervision and involvement on gaming habits, the efficacy of interventions intended to curb excessive gaming and enhance academic performance, and the long-term effects of excessive video gaming on mental and cognitive development.

## 5 STUDY LIMITATIONS

This study has a number of limitations. First, the study is cross-sectional, which collects data at a specific moment in time. Second, the sample was taken from five private schools in Jordan, which may differ from public schools in terms of socioeconomic background, access to technology, and academic demands; the results may not be entirely applicable to all Jordanian students or to students in other nations. Third, the self-reported data raises the risk of recall bias and social desirability bias, so the students may underestimate their gaming time and its effects. Fourth, another issue is the selection bias. To decrease the selection bias, five private schools were chosen at random for the study, and students were selected randomly to guarantee a representative sample of students. Fifth, even though a validated gaming disorder scale was used, measurement bias may have occurred if students misinterpreted survey items or gaming behaviour. Finally, the study did not take into consideration other possible confounders that might affect both academic achievement and gaming behaviour, such as parents’ education and socioeconomic position. These drawbacks emphasize the necessity of more long-term research with a more varied sample and impartial academic success metrics to validate these conclusions.

## CONCLUSION

In conclusion, this study sheds light on Jordanian schoolchildren's internet gaming disorder and its connection to academic achievement. The primary findings indicated that teenagers had high IGD scores, indicating a strong correlation between excessive gaming application usage and a decline in classroom concentration and attention. These results demonstrated the importance of parents’ supervision and school interventions in maintaining a balance between gaming habits and students’ academic performance and well-being.

## Figures and Tables

**Table 1:** Baseline characteristics of the participants.

**Characteristics**	Total (N= 485)	Pre-teens (N= 167)	Teens (N= 291)	***p*-value**
**Age (years), mean ± SD**	12.36 ± 2.40	11.00 ± 1.40	14.00 ± 0.90	0.001
**Gender, n (%)**				
Male	268 (58.5)	103 (61.6)	165 (56.7)	0.170
Female	190 (41.5)	64 (38.4)	126 (43.3)	
**Accommodation, n (%)**				
With family	454 (99.1)	165 (98.8)	289 (99.3)	0.630
Dorm	4 (0.9)	2 (1.2)	2 (0.7)	
**Internet Gaming Scale Score (mean ± SD)**	30.14 ± 9.28	28.20 ± 8.80	32.00 ± 9.20	0.001

**Table 2:** The characteristics of gaming application use in relation to the age category: pre-teens and teens, N (%).

**Items**	**Age Categories**			***p*-value**
	**Pre-teens**		**Teens**	
**Number of Gaming Applications Used Daily, N (%)**				
**None**	32 (35.2%)	59 (64.8%)		**0.257**
**1**	38 (41.8%)	53 (58.2%)		
**2**	38 (38.8%)	60 (61.2%)		
**3**	13 (22.4%)	45 (77.6%)		
**4**	15 (39.5%)	23 (60.5%)		
**More than 5**	31 (37.8%)	51 (62.2%)		
**Use of Gaming Applications During Class, N (%)**				
**Never**	120 (35.5%)	218 (64.5%)		**0.049**
**Rarely**	24 (45.3%)	29 (54.7%)		
**Sometimes**	14 (37.8%)	23 (62.2%)		
**Often**	8 (53.3%)	7 (46.7%)		
**Always**	1 (6.7%)	14 (93.3%)		
**Impact of Using Gaming Applications on Students` Ability to Meet Deadlines, N (%)**				
**Never**	68 (34.5%)	129 (65.5%)		**0.719**
**Rarely**	36 (34.3%)	69 (65.7%)		
**Sometimes**	45 (42.1%)	62 (57.9%)		
**Often**	13 (38.2%)	21 (61.8%)		
**Always**	5 (33.3%)	10 (66.7%)		
**Impact of Using Gaming Applications on Students` Exam Preparation, N (%)**				
**Never**	69 (35.0%)	128 (65.0%)		**0.179**
**Rarely**	43 (44.8%)	53 (55.2%)		
**Sometimes**	41 (37.6%)	68 (62.4%)		
**Often**	10 (25.0%)	30 (75.0%)		
**Always**	4 (25.0%)	12 (75.0%)		
**Impact of Using Gaming Applications on Students` Ability to Wake Up the Next Day and Attend the First Class, N (%)**				
**Never**	75 (32.8%)	154 (67.2%)		**0.070**
**Rarely**	43 (44.3%)	54 (55.7%)		
**Sometimes**	31 (45.6%)	37 (54.4%)		
**Often**	11 (31.4%)	24 (68.6%)		
**Always**	7 (24.1%)	22 (75.9%)		
**Impact of Using Gaming Applications on Students` Ability to Focus on Class, N (%)**				
**Never**	81 (34.6%)	153 (65.4%)		**0.003**
**Rarely**	33 (32.0%)	70 (68.0%)		
**Sometimes**	40 (55.6%)	32 (44.4%)		
**Often**	10 (33.3%)	20 (66.7%)		
**Always**	3 (15.8%)	16 (84.2%)		
**Frequency of Instructors Asking Students to Stop Using Electronic Games During Lecturer or Practical Sessions, N (%)**				
**Never**	116 (31.8%)	249 (68.2%)		**0.000**
**Rarely**	18 (42.9%)	24 (57.1%)		
**Sometimes**	17 (51.5%)	16 (48.5%)		
**Often**	9 (90.0%)	1 (10.0%)		
**Always**	7 (87.5%)	1 (12.5%)		
**Students' Perception of the Negative Impact of Using Gaming Applications, N (%)**				
**Never**	54 (39.4%)	83 (60.6%)		**0.364**
**Rarely**	45 (38.1%)	73 (61.9%)		
**Sometimes**	46 (38.0%)	75 (62.0%)		
**Often**	14 (25.0%)	42 (75.0%)		
**Always**	8 (30.8%)	18 (69.2%)		
**Impact of Using Gaming Applications on Students' GPA, N (%)**				
**Low**	6 (54.5%)	5 (45.5%)		**0.451**
**Intermediate**	51 (35.9%)	91 (64.1%)		
**High**	110 (36.1%)	195 (63.9%)		

## Data Availability

The data supporting the findings of the article will be available from the corresponding author [A.A] upon reasonable request.

## LIST OF ABBREVIATIONS

IGD: Internet Gaming Disorder
IGDS9-SF: Internet Gaming Disorder Scale-Short Form
DSM-5: Diagnostic and Statistical Manual of Mental Disorders, 5th Edition
ICD-11: International Classification of Diseases, 11th Revision
SPSS: Statistical Package for the Social Sciences
WHO: World Health Organization
GPA: Grade Point Average

## STANDARDS OF REPORTING

STROBE guidelines were followed.

## References

[1] Abed Alah M., Abdeen S., Bougmiza I., Selim N. (2024). From classrooms to controllers: how school closures shaped children’s video gaming habits.. Soc. Psychiatry Psychiatr. Epidemiol..

[2] Olsen M., André F., Delfin C., Håkansson A., Claesdotter-Knutsson E. (2024). Children and adolescent’s self-reported gaming habits – An exploratory, cross-sectional study of gaming among 9–15-year-old school children.. Emerg. Trends Drugs Addict. Health.

[3] (2025). Statista Video Gaming & eSports. Video Game Industry.. https://www.statista.com/topics/868/video-games/#editorialPicks.

[4] Alanko D. (2023). The health effects of video games in children and adolescents.. Pediatr. Rev..

[5] Razum J., Huić A. (2024). Understanding highly engaged adolescent gamers: integration of gaming into daily life and motivation to play video games.. Behav. Inf. Technol..

[6] Soyoof A., McLay K.F. (2019). The impact of video game intervention on reducing stress and enhancing language achievement and communication skills.. International Journal of Pedagogies and Learning.

[7] World Health Organization (2021). International Classification of Diseases, Eleventh Revision (ICD-11).. https://icd.who.int/.

[8] American Psychiatric Association (2013). Diagnostic and Statistical Manual of Mental Disorders (DSM-5).

[9] Infanti A., Valls-Serrano C., Perales J.C., Vögele C., Billieux J. (2023). Gaming passion contributes to the definition and identification of problematic gaming.. Addict. Behav..

[10] Akbari M., Bahadori M.H., Khanbabaei S., Milan B.B., Horvath Z., Griffiths M.D., Demetrovics Z. (2023). Psychological predictors of the co-occurrence of problematic gaming, gambling, and social media use among adolescents.. Comput. Human Behav..

[11] Presta V., Guarnieri A., Laurenti F., Mazzei S., Arcari M.L., Mirandola P., Vitale M., Chia M.Y.H., Condello G., Gobbi G. (2024). The Impact of Digital Devices on Children’s Health: A Systematic Literature Review.. J. Funct. Morphol. Kinesiol..

[12] AlSamhori J.F., Toubasi A.A., Jaber D.Z., Ghanem H.H., Thainat B.I., AlSamhori A.F., Kalbouneh H. (2024). Jordanian parental perception of screen time and its association with psychological distress: A cross-sectional design.. Pediatr. Neonatol..

[13] Liu L., Yao Y.W., Li C.R., Zhang J.T., Xia C.C., Lan J., Ma S.S., Zhou N., Fang X.Y. (2018). The comorbidity between internet gaming disorder and depression: Interrelationship and neural mechanisms.. Front. Psychiatry.

[14] Tortolero S.R., Peskin M.F., Baumler E.R., Cuccaro P.M., Elliott M.N., Davies S.L., Lewis T.H., Banspach S.W., Kanouse D.E., Schuster M.A. (2014). Daily violent video game playing and depression in preadolescent youth.. Cyberpsychol. Behav. Soc. Netw..

[15] Abdallat M., Al-Sanouri M., Al-Salaymeh S., Zoubi M., Barakat T., Badwan A., Alzubi A., Murshidi R. (2024). Internet Gaming Disorder and Sleep Quality among Jordanian University Students: A Cross-sectional Study.. Clin. Pract. Epidemiol. Ment. Health.

[16] Alfaleh A., Alzaher A., Alkattan A., Alabdulkareem K., Ibrahim M.H. (2024). Prevalence of video gaming disorder in Saudi Arabia: a school-based national study.. J. Egypt. Public Health Assoc..

[17] Ward M.R. (2018). “Cutting class to play video games”.. Inf. Econ. Policy.

[18] Öztürk M., Sarikaya İ. (2021). The relationship between the mathematical reasoning skills and video game addiction of Turkish middle schools students: A serial mediator model.. Think. Skills Creativity.

[19] Fatima A., Amin R. (2023). Academic Performance and Internet Gaming Disorder: A Cross-Sectional Study.. Journal of Professional & Applied Psychology.

[20] Borgonovi F. (2016). Video gaming and gender differences in digital and printed reading performance among 15‐year‐olds students in 26 countries.. J. Adolesc..

[21] Gao Y.X., Wang J.Y., Dong G.H. (2022). The prevalence and possible risk factors of internet gaming disorder among adolescents and young adults: Systematic reviews and meta-analyses.. J. Psychiatr. Res..

[22] Ferguson C.J. (2015). Do angry birds make for angry children? A meta-analysis of video game influences on children’s and adolescents’ aggression, mental health, prosocial behavior, and academic performance.. Perspect. Psychol. Sci..

[23] Alzahrani A.K.D., Griffiths M.D. (2025). Problematic Gaming and Students’ Academic Performance: A Systematic Review.. Int J Ment Health Addiction.

[24] Rajab A.M., Zaghloul M.S., Enabi S., Rajab T.M., Al-Khani A.M., Basalah A., Alchalati S.W., Enabi J., Aljundi S., Billah S.M.B., Saquib J., AlMazrou A., Saquib N. (2020). Gaming addiction and perceived stress among Saudi adolescents.. Addict. Behav. Rep..

[25] Farchakh Y., Haddad C., Sacre H., Obeid S., Salameh P., Hallit S. (2020). Video gaming addiction and its association with memory, attention and learning skills in Lebanese children.. Child Adolesc. Psychiatry Ment. Health.

[26] Al-Ali N.M., Yaghy H.S., Shattnawi K.K., Al-Shdayfat N.M. (2018). Parents’ knowledge and beliefs about the impact of exposure to media violence on children’s aggression.. Issues Ment. Health Nurs..

